# The Independence of the Sympathetic Nervous System, Demonstrated by Anatomical Researches

**Published:** 1844-04

**Authors:** 


### ART. VII.

1. *Die Selbständigkeit des sympathischen Nervensystem durch anatomische Untersuchungen nachgewiesen.* Von F. H. BIDDER und A. W. VOLKMANN, Professoren in Dorpat. Nebst drei Kupfertafeln.—*Leipzig*, 1842. 4to, pp. 88.

*The Independence of the Sympathetic Nervous System, demonstrated by Anatomical Researches.* By F. H. BIDDER and A. W. VOLKMANN, Professors at Dorpat. With Three copperplates.—*Leipsic*, 1842.

2. *Untersuchungen über den Bau den Nervensystems.* Von Dr. B. STILLING und Dr. J. WALLACH. Erstes Heft, enthaltend *Untersuchungen über die Textur des Rückenmarks.* Mit Abbildungen.—*Leipzig*, 1842. 4to, pp. 52. Zweites Heft, enthaltend *Untersuchungen über die Textur und Function der Medulla Oblongata.* Mit 7 Tafeln Abbildungen.—*Erlangen*, 1843. 4to, pp. 72.

*Researches into the Structure of the Nervous System.* By Dr. B. STILLING und Dr. J. WALLACH. Part I—*Researches into the Texture of the Spinal Cord.* 1842. Two Plates. Part II—*Researches into the Structure and Function of the MedullaOblongata.* 1843. 7Plates.

3. *Traités et Découvertes sur la Physiologie de la Möelle Epinière.* Par J. VAN DEEN, Docteur en Médecine, &c. &c. *Traduits du Hollandais, augmentés de nouvelles Recherches, &c.*—*Leide*, 1841. 8vo, pp. 224.

*Treatises and Discoveries on the Physiology of the Spinal Cord.* By J.VANDEEN, M.D. &c. *Translated from the Dutch, &c.*—*Leyden*, 1841.

4. *Untersuchungen über die Functionen des Rückenmarks und der Nerven.* Von Dr. B. STILLING, pract. Artze und Wundartze zu Cassel. Mit Abbildungen.—*Leipzig*, 1842. 8vo, pp. 316.

*Inquiries into the Functions of the Spinal Cord and Nerves.* By Dr. B. STILLING, Physician and Surgeon at Cassel.—*Leipsic*, 1842.

5. *Untersuchungen über das Nervensystem.* Von Dr. JULIUS BUDGE. Erstes Heft, 1841. 8vo, pp. 188. Zweites Heft, 1842. 8vo, pp. 238.

*Inquiries respecting the Nervous System.* By Dr. J. BUDGE. Two Parts.—*Frankfort*, 1841-2.

6. *Die Physiologie des Rückenmarks mit Berüchsichtigung seiner pathologischen Zustände für pratische Aertze.* Von BENEDICT SCHULTZ, Doctor der Medicin.—*Wien*, 1842. 12mo, pp. 70.

*The Physiology of the Spinal Cord, with reference to its Pathological States, &c.* By Dr. B. SCHULTZ.—*Vienna*, 1842.

7. *Mikroskopiske Undersögelser af Nervesystemet.* Ved ADOLPH HANNOVER, Lic. Med.—*Kjöbenhavn*, 1842. 4to, pp. 112.

*Microscopical Investigations of the Nervous System.* By Dr. A. HANNOVER.—*Copenhagen*, 1842. With Seven Plates.

8. *Untersuchungen über die Physiologie der Nervenfaser.* Von Dr. GEORG HERMANN MEYER, Privatdozenten zu Tubingen, &c.—*Tubingen*, 1843. 8vo, pp. 316.

*Inquiries into the Physiology of Nervous Fibrils.* By Dr. G. H. MEYER, Private Teacher at the University of Tubingen.—*Tubingen.*

THE anatomy and physiology of the nervous system constitute a continually widening field of research to the physician, and it is matter for satisfaction that the cultivators become more numerous as its boundaries enlarge. It would be scarcely possible to estimate this branch of medical science too highly. Practical medicine in all its branches will be efficient in proportion as our neurological knowledge becomes more accurate and extended, but especially in those departments which comprise the diseases of the brain and nerves. General hygiene will draw largely also upon neurology for first principles; the laws regarding insane men must be based upon it; and mental philosophy or metaphysics must henceforth be cultivated as a portion of the physiology of the nervous system. These remarks will suffice as an apology, if one be needed, for presenting so long a list of works on the subject to the notice of our readers.

I. ELEMENTARY ANATOMY OF THE SYMPATHETIC SYSTEM. It is not yet decided by anatomists and physiologists whether the sympathetic be an independent system of nerves, or dependent on the cerebro-spinal system. In our review of Valentin's 'Four Books on the Functions of the Cerebral Nerves and the Sympathetic,' we expressed ourselves as coinciding in opinion rather with Müller and Remak, who maintain the former, than with Valentin, who holds to the latter.* The anatomical researches of Professors Bidder and Volkmann were instituted with the express purpose of deciding the question; with what result is sufficiently indicated by the title of their publication.

The two anatomists entered on their undertaking with a full knowledge of the points to be ascertained, and with a thorough conviction of the difficulties to be overcome. They knew that unwearied industry, unflinching perseverance, and the utmost caution against errors, were necessary to a successful result; and, in accordance with these views, they have, at least in one animal (the frog), traced the whole sympathetic, twig by twig, fibril by fibril, and had so accustomed their eyes to these microscopic researches, that when a good preparation of the nervous system of a frog was submitted to their inspection, they could say, at a glance, whether it was derived from the sympathetic or cerebro-spinal system; whether from a motor or sensitive twig; or whether from the root or trunk of a nerve: nay, they could even decide from this simple inspection, if the preparation were a portion of a spinal nerve, whether it was taken from above or below the point of junction of the nerve with the sympathetic branch, or far from it or near it; and if this point of junction itself were placed under the microscope, they could say whether it belonged to the three upper, the three middle, or the three lower spinal nerves. Having so thoroughly qualified themselves for microscopic researches, they claim the confidence of their readers as to the accuracy of the researches themselves.

The first division of the essay is a view of the elementary composition of the sympathetic and cerebro-spinal system. The distinctive characteristics of the sympathetic ganglia were too striking to remain unnoticed by anatomists; as regards these they were agreed; but the relation of these ganglia to each other, to the nervous fibrils issuing from them, and to the cerebro-spinal system, gave rise to considerable differences of opinion. On the one hand, it was thought that the ganglia were like

* British and Foreign Medical Review, vol. XI, pp. 299-300.

little brains, and originated from fibrils; on the other that they were plexuses only, and that the fibrils entering them passed through them unchanged. Ehrenberg considered the microscopic characteristics of a sympathetic fibril to consist in a varicose structure, resembling strung pearls, and in a definite thickness of the primitive fibrils. This was to a certain extent a microscopic illusion; he discovered, however, the ganglionic globules. Treviranus did not confirm these researches, except that he found the primitive fibrils of the sympathetic were three or four times smaller than those of the cerebro-spinal nerves. He found, too, that the cylinder of which they were composed contained a softer matter than that of the cerebro-spinal nerves, and that the space observed between the contained matter and the outer membrane of the cylinder of the latter was wanting in the former. According to Valentin's researches, the ganglionic globules discovered by Ehrenberg are peculiar to the fibrils of the sympathetic ganglia and nerves, giving them their distinctive colour by being interspersed among them—the fibrils themselves being white. Valentin maintained also that there was no real difference between the two great classes of nerves.

The views of Remak are already, doubtless, well known to all of our physiological readers through Dr. Baly's translation of Müller's Physiology and our own analysis of them, vol. VII. Remak terms the sympathetic the system of organic nerves, and adopts Valentin's views respecting the presence of globules or small oval knots, which closely resemble the ganglionic globules, but they are *on* the fibrils, not *among* them. The colour also of the fibrils is not dependent upon these bodies, but upon the peculiar organic structure of the fibrils themselves. Hence Remak added the term gray to organic as a synonyme, and consequently described the sympathetic as the gray or organic nerves. Müller and Schwann adopted Remak's views, Valentin, on the contrary, stoutly opposed them. He maintained that the elementary structures described by Remak were only the membrane of the ganglionic globules, prolonged over the efferent fibrils, and that their softness and gray colour were caused by these membranes. The knots on the fibrils described by Remak are, according to Valentin, partly dependent on the remains of cells of the fibril-like epithelium, and partly an optical delusion. He insisted, too, that Remak had mistaken fibrils for cellular tissue. Henlè then entered the controversy in favour of Remak, acknowledging, however, that the latter had confounded various structures as epithelium, vessels, &c., with nervous fibrils. At a later period, Henlè repeated this acknowledgment more distinctly. Purkinje and Rosensthal next joined Valentin against Remak, but maintained that what Valentin called fibril-like beaded epithelium, ought properly to be termed the *formatio granulosa*, an elementary formation, having no special relation to nervous structures; and that the globules of which it consists by no means indicated the presence of organic fibrils. They insisted, however, against Valentin, that the latter really exist, and are characterized by a yellowish appearance, by their less size, their softness, and the want of double walls, and by their smoothness and but slightly granular surface. One of the inferences from their researches was that the cerebro-spinal nerves of the fœtus were not different from the permanent sympathetic nerves of the adult, and that the latter should be considered as a lower grade of development of the former. Pappenheim followed Purkinje and Rosenthal in the controversy, with whom he agreed as to the nature of the supposed ganglionic globules of Remak, and as to the similarity of the two classes of nerves in the fetal stage of development. He allowed also the existence of organic fibrils, and with Purkinje stated that they might be known from cerebro-spinal fibrils by their magnitude, contexture, and behaviour to acetic acid; and he agreed with Valentin as to the relation between the ganglionic fibrils and the nervous twigs. Pappenheim also found that a cerebro-spinal nerve never contained organic fibrils, unless when these were derived from the sympathetic nerves or ganglia, and that the branches connecting the latter with the spinal cord might be traced into the roots of the spinal nerve. Valentin, however, was not induced to change his own opinions, but maintained them the more strongly; and so our two professors step in to decide the important questions raised during the controversy, and (if that were possible) to end the strife. We shall now proceed to present our readers with an analysis of the results of their researches.

As regards the colour and softness of the sympathetic nerve. According to Bidder and Volkmann, the colour is neither dependent upon interspersed globules, nor upon the sheaths of the fibril, but is *sui generis*, and dependent upon the fibrils themselves. The nerves are softer than those of the cerebro-spinal system, because the tendinous fibres in the sheath of the latter are wanting. As regards the elementary structure, our authors agree with Valentin, Henlè, Purkinje, Rosenthal, and Pappenheim in denying the existence of Remak's organic fibrils; they are opposed to all analogy; they are only found in the nerves of the two lower classes of vertebrata, and are wanting altogether in the sympathetic nerves of the warm-blooded; they are observed as inclosing blood-vessels around which numerous twigs are distributed; they are exactly like certain described forms of cellular tissue, particularly of embryonic life, and, in short, they *are* cellular tissue. On the other hand, our authors show that the organic fibrils described by Purkinje, Rosenthal, and Pappenheim are truly described, and are not identical with those described by Remak, and that the researches directed by Valentin against Remak's views are not applicable to those of the last-mentioned microscopic anatomists. Valentin is in error respecting the true nature of the sympathetic fibrils as much as Remak. According to Bidder and Volkmann, the fibrils of the sympathetic do not exhibit under the microscope the two dark textures peculiar to those of the cerebro-spinal system. They are, indeed, included within distinct boundary-lines (grenzlinien), but these are much less dark and broad. In this respect they have a remarkable resemblance to the cerebro-spinal fibrils in the embryo. In embryo calves, from two to three inches long, Bidder and Volkmann could detect no difference between them, even with the best microscope. Towards the end of the embryonic period, the sympathetic is little altered, but the cerebro-spinal fibrils are very similar to those of the sympathetic in the adult. Bidder and Volkmann infer from observations of this kind, that the difference between the two classes of nerves is not so great as to demand their appropriation to distinct tissues.

According to observers generally, the primitive sympathetic fibril appears not as a hollow but as a solid cylinder, without the double membranous tube peculiar to the cerebro-spinal nerves. This view is confirmed by our authors. There are cases, however, in which these distinctive marks fail. In the perfectly fresh condition—that is, immediately after death—the fibrils of a cerebro-spinal nerve appear as perfectly transparent solid cylinders, with simple sharply-defined outlines (conturen), and it is only later that the two parallel lines are observed, giving the appearance of the double membranes. Occasionally this condition is observed in sympathetic fibrils. Acetic acid coagulates the contained matter of the latter, leaving the cerebro-spinal fibrils untouched; but this is a doubtful mode of distinguishing the two, and has probably given rise to numerous errors. Other uncertain characteristics are mentioned: that, however, on which Bidder and Volkmann rely is the admeasurement of the fibrils. The fibrils of both classes of nerves vary much in magnitude, as is shown by a table in which the diameter of various nerves from man, the cat, the calf, hen, frog, and pike is given. The largest fibrils of the sympathetic are, however, much smaller than the smallest of the cerebro-spinal. Part of this table we give. The figures intimate fractions of the Parisian inch: the nerve examined was the anterior root of a spinal nerve.

| | *Cerebro-spinal fibrils.* | | *Sympathetic fibrils.* | | |
|---|---|---|---|---|---|
| | Smallest | Largest | Smallest | Largest | Dimensions never observed. |
| Man . . | 0·00046 | 0·00010 | 0·00015 | 0·00020 | 0·00021 — 0·00045 |
| Cat . . | 0·00044 | 0·00077 | 0·00015 | 0·00020 | 0·00021 — 0·00043 |
| Calf . . | 0·00044 | 0·00088 | 0·00015 | 0·00020 | 0·00021 — 0·00043 |
| Frog . . | 0·00014 | 0·00070 | 0·00010 | 0·00022 | 0·00023 — 0·00043 |

The most usual size was the medium of the two measurements. The greatest variation is observed in the cerebro-spinal nerves. In frogs the smallest observed diameter of a sympathetic fibril was 0·00014 inch, the largest 0·00022 inch; while in the cerebro-spinal system, the two extremes were respectively 0·00022 and 0·00070. In the pike the extreme in the latter is still greater, namely, 0·00024 and 0·000132.

The second division of Bidder and Volkmann's treatise is devoted to a consideration of the connexions between the sympathetic and spinal nerves. In a previous essay,* Volkmann had stated, after having examined all the anastomoses of the cerebro-spinal and sympathetic, that although for the most part the sympathetic fibrils in the cerebro-spinal nerves took their course both to the centre and circumference, yet it was evident that in many places the communicating branches were exclusively peripheral. He stated also that the fibrils of a communicating branch entering the spinal cord were divided into two sets, the one turning towards the head, the other towards the pelvis. In the frog the anterior roots of all the spinal nerves communicate with the sympathetic. The first, or hypoglossal, seemed to Volkmann, indeed, to be an exception: but more accurate researches have removed all doubts. The communicating branch is scarcely distinguishable by the naked eye, and in one instance contained only two fibrils. After joining the nerve the greater portion of the fibres go to the centre, the lesser to the periphery. The

* Ueber die Faserung der Rückenmarkes und des sympathischen Nerven in Rana esculenta. Müller's Archives, 1838, p. 274.

communicating branch with the second spinal, which supplies the arm, is distributed in a contrary manner, the greater portion going to the periphery. The third spinal nerve forming with the second the brachial plexus receives comparatively few fibrils from the sympathetic: they take principally a centric direction. The greater part of the fourth communicating branch runs also centrically; but the contrary takes place in the fifth: in some instances, however, the branch divides into two equal portions for the centre and periphery, and this occurs also in the sixth. The greater part of the fibrils of the seventh, eighth, ninth, and tenth are peripheral. The tenth is a very small branch, and appears, in some instances, to be altogether absent. The connexions of the cerebral and sympathetic nerves are traced with the greatest difficulty, and no very satisfactory statements respecting them can be made. It seems, however, that the sympathetic fibrils take in general a peripheral course. Bidder and Volkmann give numerically the actual distribution of the communicating fibrils in five instances, the measurements having been made with the micrometer. The results prove what may be inferred generally from the preceding statements, that the greater portion (more than two thirds) of the fibrils in the *rami communicantes* of the sympathetic take a peripheral course. They show also that the sympathetic is an independent system, and not an appendage to the cerebro-spinal.

Passing over the estimate and refutation of Valentin's *lex progressus*, we come to the third division, which treats of the comparative numbers of sympathetic and cerebro-spinal fibrils contained in various nerves and their twigs. If a cerebro-spinal nerve be examined at its point of junction with a communicating branch of the sympathetic, the fibrils of the latter will be found to occupy one side or the other, in compact fasciculi. On tracing the further course of the nerve, these will be found to subdivide into fasciculi, and to unite with similar bundles of cerebro-spinal fibrils in a plexus-like manner, subdividing continually, even to their termination, until the fibrils of the two classes of nerves seem to be lost in their elementary constituents, and are completely confounded with one another; and this statement is applicable to all nerves. In the parts supplied with the motor and sensitive twigs the union of the terminating fibrils takes place, however, in a mode highly curious. We shall not follow our authors through the detail of their interesting and laborious researches, but content ourselves with placing the general results at which they arrived before our readers. The term, *small fibrils*, must be understood as applying to the sympathetic fibrils, and the term, *large*, to the cerebro-spinal.

"1. The nerves distributed to voluntary muscles contain very few small fibrils; on the average, about ten per cent. We have discovered no exception to this law in all the four classes of vertebrata. The composition of the vagus shows how stringent this law is; for, although that nerve consists principally of small fibrils, yet those twigs going from it to voluntary muscles are made up almost exclusively of large fibrils. The ciliary nerves of birds and the branches distributed on the cardia of calves exhibit similar proofs of its general application.*

* For the better understanding of the text, we should here state that in birds the iris is under the control of the will. It is evident also that the *ruminantia* exercise a voluntary power over the cardia and the demi-canal connected with it.—REV.

"2. The nerves distributed to involuntary muscles, whether cerebro-spinal or sympathetic, contain an immense preponderance of small fibres; in general about 10,000 per cent.

"3. The nerves of the skin and its appendages contain the small fibrils in great numbers: in general at least 100 per cent., *rarely less* than this number, and often more, as in birds and *whelps.*

"4. In the sensitive nerves distributed on mucous membranes, the small fibrils are usually five times, and sometimes even twenty times more numerous than the larger fibrils. It must not be concealed, however, that particular researches have shown that there are exceptions to this general rule. The preponderance of the small fibrils is most strikingly shown in those branches of the fifth nerve, distributed on the mucous membrane of the nose, and in the lingual branch of the hypoglossal. It is less striking in the superior laryngeal nerves.

"5. The nerves of those mucous membranes which in a healthy condition possess little or no sensibility, are made up almost altogether of small fibrils; we may therefore conclude that the nerves which go to the gullet, stomach, bowels, and urinary bladder are composed almost exclusively of sympathetic fibrils. The difference between the two branches of the glossopharyngeal nerve in the neck is highly illustrative of this fact, for the one which goes to the upper and probably sensitive portion of the throat, although containing a majority of small fibrils, contains also many of the larger; while the second branch, distributed to the inferior and nearly insensible portion of the œsophagus, exhibits scarcely any but fibrils of the former kind. . . . . It appears, then, that the small fibrils constitute the medium of the organic processes, the larger of the psychical actions, (Thätigkeiten,) and when mixed together in a nervous trunk, the one or the other kind is predominant according as the nerve is more subservient to the psychical or organic processes." (pp. 66-7.)

The inferences from these statements are obvious. All parts of the structures of organic life must be supplied with nerves of organic life, and the number of these must be in proportion to the activity of the organic processes. Bidder and Volkmann have confirmed this inference by numerous observations. Thus, in the beak of birds, and in the teeth of mammals, vegetative life is at its minimum, and so Bidder and Volkmann found the number of sympathetic fibrils. In the cutaneous nerves of birds they are very numerous; the moulting and other organic processes carried on in the skin show that the latter enjoys an activity proportionate to their number. The nerves of those parts of the skin of birds, however, which are bare or nearly so, contain very few sympathetic or small fibrils. In those distributed on closely feathered portions of the skin of the common fowl, the organic were to cerebro-spinal fibrils, as 16 to 1, in those on the less feathered skin of the head they were as 5 to 1, and in the nerves on the naked skin of the legs they were as 55 to 42. In the nerves going to voluntary muscles they average about 1 to 10, and in the sensitive nerves they vary from 10 to 1 to 14 to 1.

*On the ganglia as giving origin to the sympathetic fibrils.* It is well known that the sum of the ultimate branches of the sympathetic is many times greater than that of its trunk. The root of a spinal nerve of a frog, whether motor or sensitive, contains scarcely 2 per cent. of sympathetic fibrils; but a muscular branch contains 10 per cent., and a cutaneous twig above 100 per cent. It is very obvious that these sympathetic fibrils are not given off by the brain or spinal cord; and at this conclusion, after discussing the whole subject *in extenso*, Bidder and Volkmann arrive. Several micrometric observations made with reference to the doctrine that the sympathetic ganglia are the source of the sympathetic fibrils are then detailed, and strongly support this opinion. They found that efferent fibrils of the ophthalmic ganglia of the cat were four times more numerous than the afferent. The efferent fibrils of the cæliac ganglion in the same animal were at least three times more numerous than the afferent. The ganglia on the posterior roots of the spinal nerves of the frog give off sympathetic fibrils. The spheno-palatine and other ganglia were also examined microscopically with similar results. A general summary of our author's views, and an explanation of the plates concludes the essay.

We feel inclined to place much confidence in the observations detailed in this essay. Professors Bidder and Volkmann seem to have taken great pains, not so much to show themselves learned and deep-thinking, as truth-loving and industrious inquirers. The mathematical precision with which these observations are detailed is of great importance. It renders them the more valuable, because it will enable future inquirers to test the accuracy of their statements. We have not alluded to Dr. Hannover's researches; as we propose to notice them separately, so that our readers may themselves compare them with the preceding; and with those we are about to detail.

II. Elementary Anatomy of the Spinal Cord. It is well known that the spinal cord, like the brain, consists of white and gray substance; the latter inclosed in the former, and presenting two lateral portions, of a crescentic form, the *cornua* of the crescent being towards the middle line, and connected by a commissure of gray matter. Some continental anatomists, following Rolando, have described a line of perfectly gray matter as running along the posterior part of the posterior horns of the crescent, and have given it the distinctive appellation of the gray gelatinous substance, the other being termed the gray spongy substance. (*Substantia cinererea gelatinosa*, and *substantia c. spongiosa*, *vasculosa.*) According to Remak, the latter contains ganglionic globules; the former, corpuscles resembling the red particles of the blood in frogs. The functions and elementary composition of the spinal cord have been viewed as partly resembling those of the nerves, partly those of the brain; the cord being on the one hand a conductor, on the other an originator, of the vis nervosa. We need not here review the doctrines of physiologists respecting the structure and functions of this organ: suffice it to say, that Dr. Stilling, following Van Deen, having found the results of physiological experiments to differ from those of preceding experimentalists, and not in accordance with previous anatomical views, resolved to examine the spinal cord for himself. The more effectually to accomplish his purpose, he sought the assistance of his friend Dr. Wallach, a dexterous and experienced microscopist, the translator of Dr. Hall's publications, and well acquainted with the current neurological views. The result of Dr. Wallach's observations was, *that the hitherto-acknowledged presence of ganglionic globules in the gray matter is a microscopic delusion, and that the cord is made up solely of fibrils running longitudinally in the white substance, and longitudinally and transversely in the gray.* These results were fully confirmed by Dr. Stilling. We find, however, in the second part, this positive contradiction to the results of previous inquirers very considerably modified. The details of their united researches into the structure of the cord are given in this, the first number of their volume. Previously to the review of these we would observe, that although the medulla spinalis of oxen, sheep, swine, different kinds of birds, and frogs were examined, the cord of a newly-killed calf was found the most suitable to their purpose, and is strongly recommended by Dr. Stilling to future observers. In summer the cord was frozen, so that thinner slices could be obtained. Dr. Wallach also invented a new form of *compressorium*, for compressing the slices. The examinations of this structure, and indeed of all delicate tissues, under a compressorium is in itself exceedingly objectionable, and leads us to place far less confidence in the results of Dr. Stilling's observations than we otherwise should have done.

*Elementary composition of the fibrils of the white substance.* They appear as smooth, transparent, colourless tubes, with a double texture, (conturen;) between the two a clear space appears on pressure, and the inner membrane incloses a colourless, transparent substance, which, when pressed out, exhibits irregular granules and minute pieces that readily run together into a globular form. The matter in this state appears of a drab or brownish colour. The empty tubes, looked down upon, appear like bundles of cut straw. The membranes of these tubes are in some degree elastic; and sometimes, in manipulating the outer, one is torn open, and, by its retraction, exhibits the inner. Their varicose appearance depends partly upon incipient decomposition, and partly upon unequal pressure while being examined. When fragments of primitive tubes are separated they form into a ring, the two ends joining to each other, and easily give rise to the appearance of the granules of ganglionic globules.

*Composition of the gray spongy substance.* Dr. Wallach looked most diligently for the ganglionic globules, described by preceding observers as characterizing the gray substance. He examined it in various classes of animals, and in fetuses, and in the young as well as in the adult of amphibia, fishes, birds, and mammals. The result of his researches is, that from the decussating pyramids to the cauda equina there are no ganglionic globules to be found. They are first seen in the medulla oblongata. We find, however, that subsequently to the publication of this first part, Messrs. Stilling and Wallach were led to qualify these statements very much; and in the second part we are presented with a chapter as an appendix to this first, in which certain bodies closely resembling ganglionic globules are described as entering into the composition of the anterior gray substance of the spinal cord, and are termed by our authors spinal bodies. (Spinalkörper.) These bodies are of an angular or a star-like form, with projecting points or spines, by which means they are connected with each other and with the fibrils surrounding them. In size they resemble the ganglionic globules of the spinal ganglia, in form are altogether dissimilar; both have a nucleus and nucleolus, and contain a granular substance, but the latter are round or elliptic, and not angular and with spine-like projections. From this diversity Messrs. Stilling and Wallach conclude that the two are quite distinct. These "spinal bodies" were observed by Remak in the ganglia of the sympathetic, and it appears to us that the microscopic structures (to be hereafter described), figured by Dr. Hannover under the name of "cerebral cells," are not materially different from those described by our authors. Of course we demur at once to the name given to them. Corpuscles found in the brain and sympathetic ganglia, as well as in the cord, cannot, with any degree of propriety, be termed *spinal* bodies.

These "spinal bodies" are of various sizes, and are found in the anterior gray substance only, lying between or amongst the fibrils. They are most numerous at the points where the roots of the anterior spinal nerves pass out, and more thinly scattered towards the canalis spinalis. At many points, however, these bodies were seen in great numbers round this canal, among the fibrils of the posterior oblique commissure, and at the basis of the cornua of the posterior gray matter, but never within the substance of the latter. They are grouped in heaps like the ganglionic globules, but are not so closely pressed together.

*The gray gelatinous substance.* If the spinal cord be cut across, the outer and posterior layer of gray matter, where processes of the latter and the white substance indentate with each other, is seen to be inclosed by a substance which has a shining gelatinous appearance. This is the gray gelatinous substance. If the cord be cut longitudinally, it is seen to traverse the latter from below upwards. The continuation of this substance appears on the surface of the medulla oblongata, forming the lateral wall of the inferior portion of the fourth ventricle; it also forms the posterior surface of the medulla oblongata. This substance is composed of very minute primitive gray fibrils, which take a longitudinal direction, and separate the white matter from the gray. They appear under the microscope of a bright golden colour.

*Distribution of the blood-vessels.* The branches from the spinal arteries first pass between bundles of white matter, and then penetrate the latter, giving it a few branches. They form a fine and extensive network in the gray substance. Since the fibrils of the gray substance are as colourless and transparent as those of the white, Dr. Wallach attributes the grayness to the imbibition through the coats of the vessels of some peculiar constituent of the blood, and that this is undoubtedly connected with the functions of the gray substance and not accidental. It is evident that a greater quantity of blood is necessary to the maintenance of the function of the gray substance, than of the white.

*Intimate structure of the spinal cord.* Dr. Stilling found the fibrils of the white substance to run altogether in a longitudinal direction, and parallel with these the greater portion of the gray. In the latter, however, there were bundles of fibres running transversely and of course at right angles to the preceding, and presenting a much darker appearance than the gray longitudinal fibrils. These transverse bundles evidently dipped into the white matter, and on increasing the magnifying power, transverse fibres, hitherto unapparent, were seen to accompany the transverse bundles of gray fibrils, and extend themselves deeply between the fibrils of the white.

Dr. Stilling having frozen a portion of the spinal cord of a calf, cut off a thin slice and placed it under the microscope. It presented the following appearances. In the centre a round opening was seen—the spinal canal—surrounded by remarkably delicate fibrils of gray substance. Further, from the centre of each mass of gray substance in the cord, the fibrils are seen to diverge like the spokes of a wheel, or rather like rays from a luminous object, taking a centrifugal direction, and passing into the white substance; those from the posterior mass into the lateral and posterior strands; those from the anterior into the anterior strands. The distribution of those bundles of gray fibrils into the white substance is not the same in all parts of the cord. In the two posterior *cornua* it is very much alike, but in the anterior cords the distribution is not so regular and beautiful. The fibrils here pass from the centre as three, four, or more thick bundles, continuous with the central gray matter, and penetrate the white substance almost as far as its surface. An innumerable quantity of finer fibrils pass between these thicker bundles, (much finer, however, than in the posterior cords,) going also into the white substance, where, anastomosing with each other, they form the finest possible network.

A circular commissure was described above as consisting of remarkably delicate gray fibrils and surrounding the *canalis spinalis.* We may observe that the diameter of the latter is from 1-12th to 1-16th of a line, the breadth of the commissure about 1-10th to 1-12th of a line. In connexion with this commissure, and applied to it as tangents to a circle are two horizontal layers of fibres, situated (in man) anteriorly and posteriorly, passing from the gray substance of one side of the cord to that of the other, the fibres of which have a fan-like distribution into all parts of the gray substance. Dr. Stilling names these the anterior and posterior commissures. Their breadth averages from 1-4th to 1-6th of a line, and the length (between the two points where the fibrils begin to diverge) from a line to a line and a half. These observations of our author corroborate those formerly made by Mr. Grainger, although they differ as regards the peculiar direction or crossing of the fibres from an anterior to a posterior root. Dr. Carpenter and Mr. Newport also have shown similar transverse fibres passing through gangliated portions of the cord in the articulated, but in these the fibres are always on the same plane.

*Termination of the fibrils of the gray substance.* We have already stated that the greater part of the fibrils of the gray as well as those of the white substance take a longitudinal direction. Those of the gray, gelatinous substance are, so far as we understand Dr. Stilling, altogether longitudinal, and are traversed by *bundles* only of transverse gray fibrils in their course to the white substance. The most important problem then to solve is, how do the transverse gray fibrils terminate? Dr. Stilling examined portions of the cord in various modes with the following results. Those fibrils of the gray substance which enter into the white, after they have crossed the white fibrils in the most varied manner, run to the periphery of the white substance, and, of course, to the periphery of the cord, and there apparently form loops. These loops, however, according to Dr. Stilling, are not formed by the same fibril, nor are the two limbs of the loop on the same plane, but different fibrils running on different planes join and form the loops on arriving at the periphery; thus constituting a network of connecting loops.

The fibrils, however, of the gray matter do not pass uninterruptedly from the latter to the periphery of the cord to form the network of loops just described, for if a portion of the cord entering the line of junction of the gray and white be examined with a slight magnifying power, and under moderate pressure, the border of the gray substance consists of a wonderfully beautiful network of loops, into which very few fibrils of white substance enter, particularly in the network bordering the posterior gray masses. From this network the fibrils pass to the white substance to cross and recross with its fibrils in every direction. It is worthy of note, however, that although the two systems of gray and white approximate at so many points, and in such various modes, Dr. Stilling never saw a direct union between the fibrils of the two, or those of the one becoming continuous with those of the other.

*The connexion of the primitive fibrils with the roots of the spinal nerves.* The constituents of the spinal nerves, after traversing the *pia mater*, become connected with the spinal cord as follows:

1. Fibrils pass through the white substance, and can be most distinctly traced running deeply into the gray. For example, Dr. Stilling traced fibrils from the posterior roots to the anterior gray masses. 2. Fibrils run almost as soon as they enter the cord, between bundles of fibrils of white substance, to join other bundles of fibres from the adjoining nerves. 3. Others, in fasciculi, form loops, with fibrils coming from the next nerve. 4. Others appear most distinctly as continuations of the transverse, ray-like fibrils of the posterior gray substance; while, 5, the connexion of the anterior roots with the anterior gray substance, is still more distinct. The club-like processes of the latter are in immediate connexion with the primitive fibrils of the roots of the nerves. But, above all, it may be clearly seen that while some fibrils run directly through the white substance into the gray, others form within the former the most varied connexion with the fibrils of other roots of nerves, and cross, in every possible manner, with the fibrils of the white substance itself:—and thus it is clear how the wonderful network within the latter may be derived from the afferent primitive fibrils of the roots of the nerves, as well as from the transverse, ray-like, and, of course, efferent fibres of the gray matter. In other words, the roots of the spinal nerves may be considered as nothing more than the prolongation of the transverse gray substance of the cord; an idea of their anatomy which explains, as Dr. Stilling observes, why an enlargement of the cord is found to exist where nerves are given off.

To recapitulate. The spinal cord, according to the preceding researches, consists, first, of perpendicular fibrils, constituting the white, or cortical substance of the cord: secondly, of transverse and very delicate perpendicular fibrils, constituting the cineritious, or medullary substance, the transverse fibrils crossing at right angles to the perpendicular fibrils of both kinds, and forming a network with those of the white. Thirdly, corpuscles of an angular form, with projecting processes scattered in groups through the anterior gray matter only, but being most numerous at the origin of the anterior spinal nerves. The nerves are direct prolongations of the gray substance. The relations of the gray and the white substance vary in different parts of the cord. As a general rule, the nearer we approach the medulla oblongata the thicker are the bundles of white substance penetrating to the centre of the gray. We shall follow Dr. Stilling in the discussion of these relations, as well in the brain as in the spinal cord, in a succeeding Number. The prolongations of the gray into the white substance are more distinct in the posterior than the anterior and lateral strands, and their connexion and ramifications much less minute and numerous, for it is rare to see a bundle of gray fibres in the latter.

*Physiology of the spinal cord.* As the experimental researches of Dr. Stilling have been for the most part made to verify those of Dr. Van Deen, we can classify and compare the results of the two inquiries. To make the results more clear to our readers, we will first give them as laid down by Dr. Stilling, and afterwards review the experiments themselves in connexion with those of Dr. Budge, and with reference to the general subject. In the first place, these experiments corroborate, and render quite certain, the well-known discovery of Bell, that the anterior columns are exclusively subservient to motion, and the posterior to sensation, the experiments having been made with a careful reference to those excito-motory movements which have misled previous inquirers. We have seen that the composition of the two halves of the cord is generally the same; what, then, is the cause of this difference of function? Dr. Stilling thinks that this must be sought for in the separation and dispersion of the fibrils of the gray matter in the anterior white substance, and their complete union, even to the periphery of the posterior white substance. This is the specific difference between the two halves, and he therefore infers, that sensation is hindered by the intimate connexion of the white and gray fibrils in the anterior white substance. He thinks this view will also explain why the two layers (of gray gelatinous substance?) which separate the posterior from the lateral strands, and which are the entrance-point of the roots of the nerves, are the most sensitive of all.

The afferent and efferent properties of the posterior and anterior divisions of the spinal cord are also further established by Stilling, Van Deen, and Budge. But it does not appear that the white substance is the conductor of the stimulus. According to Dr. Stilling, if the whole cord be cut through, so that the posterior white substance only be left untouched, sensation is annihilated in the parts of the body below the section; but if, on the other hand, the posterior white substance only be divided, sensation continues (although perhaps not so vividly,) in the parts below the section. It is true that if the posterior white substance be irritated with a needle, the expression of pain is much more decided than when the gray matter of the surface of the *divided* posterior column is irritated. The anatomical arrangement of the fibres shows, however, that the gray fibrils are much more likely to be irritated when in their undisturbed connexion in the white substance, than in the destroyed gray matter. Dr. Stilling, therefore, comes to the conclusion, that the longitudinal fibrils of the posterior gray substance conduct sensory impressions from the periphery to the brain,—a proposition in perfect accordance with all the results of experiments.

What is the use of the circular commissure? It contains no decussating, but only parallel fibres, nor do the adjoining portions of the transverse commissure. From a consideration of these facts, Dr. Stilling has been led to suppose, that these structures serve as conductors of impressions from one side of the cord to the other. That there is some such bridge of communication appears certain from the results of Van Deen's experiments (corroborated by Stilling), that when one lateral half of the cord only is cut through, the parts below are deprived neither of sensation nor voluntary motion. Again, if one lateral half be divided to the median line, at one point, and the other lateral half be divided at a second point, higher or lower than the first, sensation may be still communicated, and reflex and voluntary movements excited.

Do the longitudinal fibrils of the anterior white substance conduct the influence of the will? No—this is the office of the longitudinal fibres of the anterior gray substance. If in a living animal a section of the former only be made, volition can still be directed to the parts below; but if the section be prolonged deeper, so as to divide the latter, voluntary movements at once cease.

What is the office of the transverse fibrils of the spinal cord? They cannot serve as conductors either to or from the brain, for then all must be joined with each other, and thus a continuous line would be formed many thousands of miles long. Besides, when the white substance is cut through, many of these fibres are divided, and yet volition and perception still remain connected with the parts below; so that it is clear their perfect continuity is not *directly* necessary to either the one or the other. We have, seen, however, that these transverse fibrils are prolonged into the nerves, and as we know that the posterior nerves are necessary to sensation, Dr. Stilling thinks the posterior transverse fibres must be looked upon as excitors of the posterior longitudinal fibres of the gray substance. Thus a sensation is called forth *mediately* by the transverse fibres of the posterior gray substance, and produced in the sensorium *immediately* by the longitudinal fibres. *Mutatis mutandis*, the same relations may be given to the anterior gray fibres. As centripetal or efferent impressions pass from the sensitive nerves along the transverse fibrils to excite the longitudinal, and to be thence conducted to the brain, so centrifugal or efferent impressions may pass in a contrary direction, that is, from the brain along the anterior longitudinal fibrils of the gray substance and traverse the transverse fibrils on their course to the roots of the motor nerves. Of course, in the anterior column the longitudinal fibrils of the gray substance are the excitors of the transverse, an arrangement opposed to that supposed to exist in the posterior column. In support of this view, Dr. Stilling refers to his experiments (to be noticed hereafter), and very plausibly observes that it will explain how *sensations* and their dependent phenomena, as secretion, &c., are excited at will; the sensatory influence passing along the longitudinal fibrils of the posterior gray substance, just as the volitional influence traverses the longitudinal fibres of the anterior gray substance.

Dr. Stilling having developed, as he thinks, the mechanism of ordinary sensation and motion, proceeds to consider the origin of those "involuntary movements which are known under the improper name of reflex action." Our readers will recollect that transverse fibrils pass directly from the posterior to the anterior gray substance. The conclusion from experiments, that excitation of the former by a mechanical as well as by volitional stimulus may induce excitation of the latter, and so movements follow, is evidently more fully established by this anatomical fact, as showing the track along which the stimulus passes. We can also, according to Dr. Stilling, understand how emotional excitants, as fear, may act through those connecting transverse fibrils, and excite not only involuntary movements, but also excentric sensations, as formication, weakness of the limbs, &c.

The coordinated movements and sympathies, and irradiation of sensations, are readily to be explained by the commixture and intimate union of the various fibres described, and constituting the elaborate network or plexus in the anterior and posterior white matter, we have repeatedly mentioned. Two problems remain unsolved, and which are left by Dr. Stilling for future consideration. The one is, why the longitudinal fibres of the anterior and posterior gray substance are devoted exclusively to motion and sensation; the other, why the conduction of the anterior and posterior nerves is exclusively centrifugal and centripetal.

Van Deen's Experiments. The publication of Van Deen contains: 1. An essay on the anterior and posterior columns, in which experiments are detailed establishing the doctrines of Sir C. Bell. 2. A treatise containing some recent discoveries of the properties of the spinal cord, and particularly of a nervous circulation in that organ. 3. Supplements to this treatise. 4. A translation of Kuerschner's essay on the Functions of the anterior and posterior Columns, with a criticism by Van Deen.—The second essay and supplements alone demand our notice. As we cannot give these experiments in detail, we shall select the most important: and first, we would observe that Van Deen deduces from them the general principle, that a true circulation exists in the functions of the spinal system of nerves. Impressions are received by the posterior columns and transmitted to the brain, giving rise to sensation; and the animal excites movements—*voluntary* movements—in proportion to the energy of the sensation. If the impressions received by the posterior columns are not transmitted to the brain, but directly to the *anterior* columns,* true sensation is not excited but movement only. The movement is of course reflex, and the impression thus received and acting in the spinal cord is termed by Van Deen reflex sensation—"*Le sentiment de réflexion.*" He proves by experiments that both reflex sensation and motion may be excited after division of both the anterior and posterior white or (as Van Deen terms it) medullary substance; and others are detailed (too long to describe) showing that the reflex sensation is not received by the posterior white matter, and that the anterior white matter alone is equal to the transmission or communication of reflex movements. So that reflex sensation and motion are developed and transmitted by the gray substance.

We shall now detail one or two of the numerous experiments instituted by Van Deen, and from the observation of which his views are deduced. The first experiment we give at length, as it exhibits the mode in which he prepared those unfortunate subjects of his researches, in which strychnine was made use of as a sort of physiological microscope, to develop more clearly the function of the motor track.

"Exp. i. The abdomen of a frog was opened, the abdominal viscera removed, and the vascular connexions of the spinal cord destroyed, so that from the second vertebra to the lower part of the abdomen nothing was left except the bones, muscles, and nerves proceeding to the lower extremities. The spinal cord was then opened carefully in front through the third vertebra, the anterior column divided, and all the blood-vessels destroyed which could connect the anterior with the posterior parts of the body. In this state, one or two drops of a concentrated solu-

* Our author uses the term *posterior* columns instead of anterior, but this is evidently a clerical error.—Rev.

tion of acetate of strychnia were dropped into the mouth, and in a few minutes, the following symptoms were observed: The parts above the section through the anterior column were affected with tetanus, those below were free. If, after the tetanic spasm had subsided, one of the hind feet was touched, but very slightly, reflex movements took place in the posterior part of the body, tetanus in the anterior. But if the head or a part of the surface anterior to the section was touched, tetanus came on in the same parts, but no movement in the posterior portion of the body." (53-5.)

Exp. XXV. If, in the region of the third vertebra both the anterior and posterior column of one side (say the left) be divided, and of course the gray matter lying between them, the sensation of the left hind-foot is not destroyed, for if it be irritated, the animal will exhibit signs of pain moving first the fore-feet and then the right hind-foot, and subsequently the left. The motion of the latter, however, Van Deen subsequently attempts to show is only reflex.

Exp. XXVII. If the spinal canal of a frog be opened from the second to the sixth vertebra, the left lateral half cut through in the region of the second vertebra just below the fore-foot, and the right half lower down in the region of the fifth vertebra, it will be seen that the hind-feet are deprived of all voluntary motion, but that the voluntary movements of the fore-feet excite reflex movements in the posterior extremities, particularly the left hind-foot. But further: if the hind-feet be irritated, and particularly the left, the animal clearly expresses the feeling of pain. Again, if the vascular connexion be completely destroyed in the mode previously stated, and a little strychnia be placed in the mouth, tetanus is shortly seen to affect the anterior extremities instead of the posterior. If, after the spasm has ceased, the fore-feet be slightly touched, tetanic spasm affects one hind-leg, reflex movements the other; but on the contrary, if the hind-feet be touched, tetanus is excited in both. The same results follow if the frog be not poisoned, but only decapitated.

These are one or two of the more important and interesting of Van Deen's experiments, but we must add, that we are obliged to leave others quite as ingenious altogether unnoticed. The next points Van Deen seeks to establish are, 1, the existence of a circulation through the nerves, or of a nervous power, *circulatio nervea, seu vis nervea:* the latter term is preferred by our author: and 2, that the nerves are not prolongations of the white substance of the cord. These views are but a repetition of those previously stated.

In the first of the two supplements which follow the treatise just analysed, Van Deen explains and elucidates the experiments already described; and in the second details additional researches. He also discusses the opinions of Bell, Majendie, Carus, Kronenberg, Valentin, Volkmann, Müller, and Hall. Van Deen, although acknowledging and further elucidating the physiological theory of the latter, maintains that his anatomical views are erroneous, and that the experiments detailed prove that the excito-motory system of nerves has no existence, neither in the ganglionic or cerebro-spinal system. The ganglia of the former differ from those of the latter only in being connected with the brain mediately by communicating fibrils, while the gray substance of the ganglia of the spinal cord, as in direct contact with the brain. The spinal cord in a frog may be changed into a series of ganglia, by removing at given points the whole of the gray matter, leaving only the posterior white substance in connexion with the brain. This supplement ends with a résumé of the author's views, respecting the functions of the spinal cord as deduced from his vivisections, which, in justice, to Van Deen, we give at length.

"1. The anterior white substance of the anterior columns is subservient to motion only. 2. The anterior columns with their gray substance are subservient both to sensation and motion. 3. That the white substance of the posterior cord is subservient to sensation only. 4. The posterior cords with their gray substance are also subservient to sensation only. 5. The white substance of the posterior columns need not reach to the brain to communicate there the impressions received by the posterior roots. 6. The white substance of the posterior columns, when isolated, does not readily transmit sensation. 7. This can only take place when it is in contact with the gray substance. 8. The anterior white substance, detached from the gray, cannot communicate volitional impressions along the anterior roots directly to the muscles; it can only excite vibrations in them. 9. The conditions necessary to true sensation are also necessary to reflex motion, that is to say, as the former is dependent on the posterior roots and cords, and on the gray substance, so also reflex sensation is dependent on the same structures. 10. Those conditions necessary to the transmission of true sensation along the anterior [posterior*] cords are also requisite to transmit reflex movement along the same cords in the direction from the spinal medulla to the brain, *neither the one nor the other can be effected without the gray matter.* 11. The gray substance communicates impressions from the posterior to the anterior columns, and 12, from one centripetal fibril to another. 13 This is also true of the centrifugal fibrils; for few of the fibrils of the white substance are able to transmit by means of the gray substance the volitional impressions of the brain to almost all those anterior roots which derive their origin from that part of the spinal cord corresponding to these few fibrils. 14. That the centrifugal and centripetal fibrils ought to be considered as conductors, and the gray substance as the active centre of the nervous system." (pp. 199-200.)

Stilling's Experiments. The important doctrines deduced by Van Deen from his experiments, (of which we gave his own summary in our Twelfth volume, p. 514,) and the boldness and apparent exactitude of the latter, struck the notice of Dr. Stilling, a practitioner of Cassel. He undertook the important and laborious work of repeating them; and in the work before us has reviewed them in detail, giving also a description of the mode which he adopted to verify them. With very few exceptions he declares that the experiments of Van Deen are erroneous, or, as he expresses it, false, and that consequently his doctrines are in many points erroneous. The inferences numbered 1, 2, 3, 10, 12 are utterly objected to by Stilling, and several of the remaining admitted only with limitations. So far as a perusal of the two authors enables us to decide, Stilling makes out a better case than Van Deen, yet we think with a great deal of unnecessary harshness of expression, and occasionally with an apparent exactness and positiveness which are somewhat suspicious. The correct performance of experiments so complicated as those detailed, and upon structures so minute as the divisions of the cord in the frog, can only be attained by an incredible degree of dexterity; and although both our physiologists claim credit for this dexterity, having operated upon many hundreds of unfortunate frogs, we are by no means ready to admit their claim to its full extent. Although Stilling, having had the ingenuity and experience of Van Deen to assist him,

* Anterior here must be a clerical error.—Rev.

makes out apparently a better case, we must see his researches corroborated by at least one competent witness before we can officially give our judgment against the Dutchman. The whole of Van Deen's experiments with strychnine seem to be fairly invalidated, so far as they refer to the point aimed at; because, firstly, his mode of preparing the animal does not arrest the circulation within the cord; secondly, the transmission of the poison from one part to the other takes place by imbibition, a small piece of connecting cord sufficing; and thirdly, this imbibition is influenced by gravity. Stilling observed the phenomena almost exactly as described by Van Deen, if the animal was placed on its feet and belly; but if laid on its back, or if its hind-feet be raised higher than its head, so as to hinder the circulation from the anterior to the posterior half, the results are very different indeed: and this fallacy runs through all his experiments. Again, in the experiments to elucidate the functions of the anterior white matter, Stilling contends that Van Deen irritated the *roots* of the anterior nerves, which are microscopically small in the frog, and not the *substance* only of the cord. Many of the other results of Van Deen's experiments are flatly contradicted, as we have before observed. Those in which strychnine was used as the physiological magnifier of spinal action, are the most interesting of the experiments detailed by Dr. Stilling, because they throw light as well upon the nature of the capillary circulation in the spinal cord as upon its proper function. The tenacity of life possessed by frogs is extraordinary. Dr. Stilling prepared a great number in the mode described by Van Deen in exp. i, which, when let go, would jump about with the greatest activity and ease, so that no one would suspect that the viscera and large blood-vessels of the animals had been entirely removed, and nothing left but the osseous, muscular, nervous, and capillary systems. Generally, in about half an hour, the animal leaps with less energy when irritated, shuts its eyes as if slumbering, and the respiration becomes less and less frequent, until at last death is imminent, and movements are no longer excited by a stimulus. In many frogs, Dr. Stilling has excited reflex movements a full hour after evisceration, according to Van Deen's method, by applying acetic acid to the skin. It is a necessary inference that the cord may continue to perform its functions for at least from half an hour to an hour after the general circulation of the blood is destroyed.

If the spinal cord of a frog, thus completely eviscerated, be exposed, and touched with a few drops of a solution of acetate of strychnine, at one point only, in five minutes general tetanus occurs, just as if the strychnine had been taken into the system in the usual way. If the slightest bridge of communication be left between two divided portions, the effect is the same, and transmission takes place contrary to the force of gravity; as for example, from the lower part of the cord to the upper. This, as is shown by subsequent experiments, is not due to imbibition only, for when the blood-vessels are so destroyed that all vascular communication between the upper and lower portion of the cord is cut off, the lower end still exercises its functions as well as the upper. From this Dr. Stilling argues, that there is a continuous capillary network within the parenchyma of the cord. An isolated piece of the cord will also react to strychnine, in the same way as the whole. Thus, if that portion in connexion with the fore-feet be entirely separated, above and below, from the rest, so that all communication with the head, or inferior extremities, be cut off, and then touched with a solution of strychnine, the fore-feet become tetanic. Dr. Stilling shows, that each portion of the spinal cord is a complete apparatus in itself, independent of the brain, or the other portions of the medulla, and able to transmit influence along the motor nerves in connexion with it; that, in fact, the cord may be divided into as many complete portions as there are pairs of motor nerves,—an opinion, by the way, dwelt on by Van Deen. Dr. Stilling fixes the seat of tetanus (which he terms " a paroxysm of reflex movements,"—Ein Sturm von Reflex-bewegungen,) in the anterior gray substance.

Dr. Stilling made various sections of the spinal cord and brain of frogs. The whole of the cerebral axis was divided into two equal lateral halves, by a longitudinal incision carried along the median line from the cauda equina through the cord, the cerebellum, the corpora quadrigemina, and the brain. In ten minutes after the operation, reflex movements may be excited, but they are confined entirely to that side to which the irritation is applied; no stimulus whatever can induce them in the opposite side. If the cerebellum (or the striæ that are the analogue of it,) and the brain be left undivided, and the cord be bisected along its whole length, irritation of a hind-foot will excite action in the abdominal muscles of the same side. If one or other nostril be irritated with a couching needle, the fore-leg of the same side is immediately directed to the part, and energetically endeavours to remove the irritating instrument; and when Dr. Stilling has forced the mouth open, to drop in a solution of strychnine, the animal has attempted to remove his finger with both fore-paws, but the action of the two paws was not harmonious as in unmutilated frogs. If the fore or hind-feet were irritated, flight was attempted with all parts of the opposite, as well as of the same side. A frog, with its spinal cord thus divided, strenuously attempted to escape from the needle which it saw ready to irritate it, and, in its violent efforts, brought the left hind-foot so far forward, that the web covered the side of the creature's head. From this and other statements in the books of both Van Deen and Stilling, we infer that sad cruelties have been practised on these beautiful, and (if these experiments be truly detailed,) intelligent animals. The physiological conclusion is, that the combined movements, requiring the action of the two sides, are rendered inharmonious, unless the irritation applied to one side is sufficient to reach the uninjured brain, and so pass to the opposite. It is subsequently stated in a note, (p. 128,) that if the central axis be unequally divided along its whole length, the muscular action is the stronger on that side on which the lateral half is the larger, so that the body of the animal forms a half circle, as in pleurosthotonos; or, if the cord be unequally divided at one part only, then the corresponding limbs are respectively weaker and stronger.

Van Deen's xxvth experiment, which we have shortly detailed, is important, as proving the transverse transmission of motive and sensitive influence. Dr. Stilling's repetitions of this experiment confirm that part of Van Deen's inference which relates to this point; Dr. Stilling insists, however, that the motions referred to are not reflex, but voluntary. He also attempts an explanation of this new fact in physiology, by supposing that the volitional impulse is excited upon the spinal cord generally, and from thence to special muscles along the anterior nerves. For the proper distribution of this impulse he infers from his experiments, (and it must be remembered, as of importance in estimating the value of his anatomical researches, that this theory preceded the latter,) that there is a peculiar anatomical arrangement, of such a kind that the one half of the cord is able to receive the excitation of the other, the upper portion that of the lower, and *vice versâ*. By this relation of the various portions of the cord to each other, we can understand the co-ordination of movements. The same doctrine applies to sensation. In this, the xxvth experiment of Van Deen just alluded to, the irritation applied to the right hind-leg passes through the right posterior roots into the right lateral half of the cord, and through the posterior into the anterior white and gray substance: the latter communicates the impression, at the same moment, to the left lateral half, above the transverse section, and from thence upwards, in both sides of the undivided cord. Experiments xxvi, xxvii of Van Deen, are only modifications of the xxvth; each half of the cord is divided at *two* points distant from each other, (opposite the second vertebra on one side, opposite the fifth on the other,) with similar results as to the transverse transmission of both volition and sensation. In his xxviiith experiment, Van Deen makes two sections in the opposite halves of the cord, but only a line or two apart, and shows, that in this case, no transmission of volition or sensation across the cord takes place. This inference Dr. Stilling flatly contradicts, and almost insinuates that Van Deen can never have made the experiment; not unbiassed in this, mayhap, by his own little theory of that "peculiar anatomical arrangement," in virtue of which a very little connecting link of spinal cord is a sufficient bridge for volitional or sensory impression to travel over. If energy in protestation will convince, Dr. Stilling is very convincing.

The next series of experiments are intended to illustrate the functions of the white and gray matter in the anterior and posterior strands. The posterior white matter in the region of the third vertebra of a frog was completely removed, with the greater part of the posterior gray matter; the hind-feet of the animal still exhibited both sensation and power of voluntary motion. The posterior white and gray matter was entirely removed in the same mode, together with a portion of the anterior gray matter; the hind-legs were still capable of reflex and voluntary movements, but utterly insensible to any stimulus. A portion of the spinal cord was removed, as in the preceding, so that only the anterior white substance remained to connect the upper and lower portions of the cord, all the gray matter, and the posterior white matter, being completely removed: the hind-feet were utterly incapable of sensation or voluntary motion, or of reflex movement, when the irritation was applied to parts above the injury, as the head or fore-feet. When the hind-feet, or anus, were irritated, reflex movements are excited in the former, but not in the anterior extremities. One or two other experiments of this kind are shown to exhibit the same results. Dr. Stilling makes the interesting remark, that the animal which has been thus deprived of the use of its hind-legs, makes strenuous efforts to move forwards with its fore-legs, and exhibits all those muscular movements in the eyes, neck, &c., which indicate intense suffering. He takes occasion to compare the general muscular acts of a man who is putting forth his whole strength with those which express pain, and shows the great similarity of the two; an analogy not without an important application to the theory of reflex acts. This part of the book closes with an experiment, from which, in connexion with the preceding, Dr. Stilling infers, " that so long as the continuity of the gray substance of the cord, and of its connexions with the roots of the nerves is unbroken, the white substance may be cut through, both on the anterior and posterior surface of the cord, whether at points close to, or distant from each other, without either true sensation or voluntary motion, reflex sensation (sensation of the cord after the removal of the brain,) or reflex motion being thereby abolished." (p. 197.)

The next section of Dr. Stilling's book is devoted to a criticism on Van Deen's explanation of his experiments. Tigers are forbidden to devour tigers, so we will leave this criticism in possession of all its weight with those who may read the original. The discussion is, for the most part, theoretical; and we suspect our physiological readers can theorize for themselves, as well as either of our authors. The section closes with a hypothetical explanation of the anomalies developed in comparing reflex and voluntary movements, and Dr. Stilling infers that there must either be two classes of motor and of sensitive fibres, or that different parts of the cord have different functions. The former, he says, have not yet been anatomically demonstrated, we therefore take this opportunity of directing his attention to the researches of Mr. Newport and Dr. Carpenter, reviewed in our last Number, p. 171.

Van Deen's theory of a nervous circulation is next criticised and opposed. Dr. Stilling here refers to the action of flexion and extension, an important class, respecting which we as yet know little. He views extension as a voluntary, flexion as a reflex act; the one excited by the brain, the other by the cord, in which it must be performed, or specially favoured, by a suitable anatomical arrangement of the fibres. Dr. J. Budge has come to a similar conclusion, though we suspect in his case, as in Dr. Stilling's, the theory has developed the experiments rather than the experiments the theory. In opposition to Van Deen's views, Dr. Stilling maintains that every impression on the cord, whether it be volitional or sensory, acts on the whole mass of the cord at the same moment, and only allows that particular points or divisions of the cord may be more strongly impressed than others. For example, he maintains that in making any volition efforts, even the slightest, as the act of writing, there is a certain amount of tension in the whole cord, and, of course, in the whole muscular system, although that tension is greatest in that part of the cord with which the muscles of the head and arm are connected. Is it possible that in certain combined mechanical acts of flexion and extension, as perching, flying, &c., there is an alternate state of action and repose in two opposed structures of the cord, just as there is in the heart, the respiratory muscles, &c.? In this way we may explain many unexplained phenomena. These views of Van Deen and Stilling are applicable to various pathological states of the nervous system, especially to centric structural changes; but these applications we must leave to our readers, and close our review with Dr. Stilling's own résumé of his views. We would first, however, observe that he is much more indebted to Van Deen than he is willing to acknowledge; that he has profited largely by the ingenuity and experience and (we must add) mistakes of the latter; and that in fact the impulse to his researches, both anatomical and physiological, is derived from the Dutchman.

"1. The posterior roots are sensitive and not motor. 2. The anterior are motor and not sensitive. 3. The posterior white substance is sensitive when in connexion with the posterior gray matter, otherwise it is not. 4. The posterior gray matter is sensitive whether in connexion with the posterior white matter or not. 5, 6. Neither the anterior white nor gray matter are sensitive whether in connexion or separate. 7. Motion takes place, specially through the anterior gray matter, whether it be voluntary or reflex .......... 8. The anterior white matter receives impressions from the anterior gray matter, communicates them to the roots of the anterior nerves, and so develops motion, whether voluntary or reflex. 9. The posterior white substance receives impressions from the roots of the posterior nerves, communicates them to the posterior gray substance, and thus the latter is the medium of sensation. 10. Sensation takes place solely through the posterior gray substance, and never without its agency. 11. The posterior as well as the anterior white substance acts across the cord and not longitudinally; the one conducts *to* the axis of the cord, the other *from* the axis towards the circumference. .......... 12. The influence of the will always acts on the whole mass of the spinal cord, but stronger on some points than others. The development of that influence, however, takes place solely through the anterior gray substance. 13. Every sensory impression (empfindung) acts on the whole spinal cord, but stronger on some parts than others; this development occurs solely through the posterior gray matter. 14. So long as a small portion of the posterior gray matter connects the lower portion of the cord with the upper portion and the brain, sensation remains unchanged in the inferior extremities. 15. And so long as a small connecting bridge of anterior gray matter connects the upper and lower portion of the cord, so long voluntary motion remains, more or less perfect, in the inferior extremities. 16. The same laws apply to reflex sensation and reflex motion as to true sensation and voluntary motion. 17. Reflex movements and the coordinate movements accompanying volition, have both their origin in the peculiar anatomical arrangements of the spinal cord. 18. The coordinate movements may occur during primary reflex movements, excited through the sensitive nerves, without the cooperation of the roots of the sensitive nerves, or of the posterior white and gray substance. Neither is the presence of the posterior roots necessary to the propagation of the reflex change to parts of the cord not originally excited. 19. The mode in which impressions are communicated from the periphery to the spinal cord and brain, as well as the mode in which the will acts from the brain on the spinal cord and nerves, is quite unknown, and all notions of a circulation of nervous principle are founded altogether on deception and error. 20. The gray matter of the cord is the proper and principal agent from which the white substance derives its powers. Sensation and motion are both derived primarily from the gray matter, and the white substance forms, modifies, and gives their peculiarities both to sensation and motion. The nerves only act as conductors to the spinal cord." (pp. 305-10.)

The plate attached to Dr. Stilling's work contains a demonstration of the mode in which the spinal column of frogs should be opened, and also figures illustrating the various sections of the spinal cord after Van Deen. Dr. Stilling's descriptions and explanations of his experiments are intelligible and precise, contrasting favorably in these respects with Van Deen's.

The Experiments of Dr. J. Budge. The first part of Dr. Budge's publication came out when he was a practitioner at Altenkirchen, a town situate in a wooded, mountainous district, from which he was supplied with many hundreds of mammals and birds for his vivisections. Subsequently he became more ambitious, for on the cover of his second part we find him announced as a "privat docent" at the University of Bonn. His style and the subjects of his experiments evidently alter with his circumstances; frogs constituting the principal of the latter, and an assumption of profundity the former. According to his plan, a third part is to follow. We shall condense the results of his experiments so far as they refer to the anatomy and physiology of the sympathetic and spinal system, leaving his researches on the encephalic organs and his theories for future discussion.

*Arrangement of the sensitive and motor fibres of the spinal cord.* The vertebral column of various living animals was opened, and the spinal cord irritated and sliced in various ways. The results are the following:

No portion of the cord is without sensibility; in its whole breadth, its whole length, anteriorly, posteriorly, laterally, and centrally, everywhere, there is sensibility.

The sensibility of the outer layer of the posterior part of the cord is much greater than that of the anterior surface, but the latter being removed, the sensibility of the two is about the same.

The sensitive fibres remain on the same side, along the whole length of the cord, the right on the right side, the left on the left side.

Motor fibres run through the whole thickness of the spinal cord, anteriorly and posteriorly; in short, through its whole circumference.

Although the motor fibrils of the whole body are collected on the anterior surface of the cord, and, consequently, motions appear more vigorous after irritating these, yet the capability of movement is removed by destruction of the posterior strands as well as of the anterior. (p.15.)

All the fibrils of motor nerves are directed from without towards the middle line of the cord, and from without fresh fibrils are prolonged until they decussate with those from the other side of the cord; those of the lower extremities decussating in the medulla oblongata of the upper extremities in the pons Varolii. (p. 27.)

The motor fibrils which are subservient to extension are situate in the anterior column, and decussate the fibrils subservient to flexion, situate partly in the posterior partly in the anterior column, the central ends being in the former. (p. 47.)

Budge maintains, further, that the fibrils of the extensors and flexors of different groups are situate together, just as the corresponding muscles; the flexor fibrils of the right leg lie together in one group, those of the left leg in another, and so also with regard to the extensors. Budge is also of opinion that the anatomical arrangement which determines the coordinate movements is seated in the cord.

An experiment on a dog is detailed, in which part of the dorsal portion of the spinal cord was divided, leaving only a connecting portion of anterior white matter, the whole of the gray being entirely cut through yet sensation remained, though imperfectly in the lower extremities. Budge infers, consequently, that there are a few sensitive fibres in the anterior white matter. We are of opinion, however, that much value cannot be attached to this and several others of his experiments. He confirms, however, the views of Van Deen and Stilling as to the transmission of sensation *across* the cord. Some of the experiments of both these physiologists are repeated and different results obtained. For example, Budge infers that there are no distinct portions of the spinal cord traversed by the will, nor any fibres specially adapted to the communication of it to the roots of the nerves. There are only sensitive and motor fibrils in the cord, with ganglionic globules in different stages of development.

*Physiological relations of the sympathetic.* Budge made numerous experiments to ascertain the physiological relations of the cerebro-spinal to the sympathetic system. He opened the thoracic and abdominal cavities, and, irritating various parts of the nervous centres, watched what viscera were excited.

*The heart.* If the medulla oblongata of a frog be exposed, and, the heart having ceased to beat, a needle be thrust into the anterior cords at the upper end, directly in the middle line, and moved up and down, the heart will begin to beat again; sometimes the auricles only, often both the auricles and ventricles will act. Irritation with *kali causticum* produces the same result. The medulla oblongata and cervical portion of the cord was exposed in a cat: it died immediately, but while the heart was beating slower and slower—once only in fourteen seconds—the posterior cords were irritated without result; but so soon as the anterior columns were excited, the contractions increased to one in three seconds. As in this experiment the anterior gray matter appears to have been the origin of the increased motion, it is interesting, as showing that that portion of the cord assists in some, at least, of the involuntary movements as well as the voluntary. Budge's conclusion from his experiment is, that the motor nervous fibrils of the heart lie near the middle line, and in the anterior strands of the cervical cord and medulla oblongata; and that irritation of no other portion of the nervous centres will re-excite the heart's action. In his second part, however, he finds, from subsequent experiments, that irritation of the corpus callosum re-excites the cardiac movements.

As regards the viscera, Budge found that irritation of the cerebellum excited motion of the stomach; irritation of the anterior column of the cord, the corpora striata and quadrigemina, and the optic lobes re-excited the peristaltic of the small intestines; irritation of the cerebellum, and also along the whole length of the anterior column of the cord, and of the same structure, especially at its point of junction with the medulla oblongata, induced vivid contraction of the colon and of the urinary bladder.

In the first part various experiments are detailed, which show that irritation of one side of the cerebellum—for example, the left—caused the right testicle to be raised up, so that the spermatic cord formed nearly a right angle on itself. The uterus, fallopian tubes, and ductus deferens have a similar connexion with the cerebellum. In the second part we find the result of these experiments confirmed, with this exception, that irritation of the cerebellum excited movements in the organ of the *same* side as well as of the opposite. Perhaps we ought to observe here that Bidder and Volkmann irritated the third communicating branch of the pike, the fibrils of which are distributed peripherally with the spinal nerves, and movements of the anal fins were induced. The preceding results are all we shall present to our readers from Budge's publication. We may state, however, that other matters are discussed at length; as, for example, the nature of voluntary motion, and of a power seated in the cerebellum termed by him "Hemmungskraft," which restrains or counteracts the action of the nervous centres on the muscles. The involuntary respiratory movements, and the nature of sensation and reflex motion are also considered at length. Dr. Budge concludes, with respect to the latter, that there are no reasons for believing, with Dr. Hall, in the existence of excito-motory fibrils distinct from those of true sensation and volition. There are only sensitive and motor fibres in the spinal cord. He also thinks that the latter is not the only organ in which reflex motions are developed. (Heft ii, pp. 232-5.)

The brochure of Dr. Benedict Schultz consists of two divisions, and seems to be the performance of a young physician. In the first we have a good summary of the modern physiology of the spinal cord, as well as its pathological applications. The views of Müller, Hall, and Stilling are examined at length, as to the excito-motory system, and our author, like most of his countrymen, takes the opportunity of doubting the anatomical existence of Dr. Hall's excito-motory nerves.

In the second division we have a notice, translated from the Polish language, of a paper communicated to the medical professors of the Iagellonic University at Cracow, and published by them in their Annual Transactions. It appears that the faculty possessed by dilute acetic acid of rendering certain animal tissue transparent has enabled Purkinje to discover a distinct nervous tissue in the pia mater and dura mater of the spinal cord, composed of numerous fibrils, analogous, apparently, to those of the sympathetic system.

Our allotted space being occupied, we shall notice the other works named at the head of this article in our next Number.

---

